# Synergistic and antagonistic AML cell type-specific responses to 5-AZA-2’-Deoxycytidine and 1-H-D-Arabinofuranosylcytosine

**DOI:** 10.1186/1471-2164-15-S2-P28

**Published:** 2014-04-02

**Authors:** Abeer Abd Elmoneim, Elisabeth Heuston, Daniel H Wai, Timothy Trich, Robert J Arceci

**Affiliations:** 1 Paediatric Department, Sohag University, Sohag, Egypt; 2 Sidney Kimmel Comprehensive Cancer Center (SKCCC) Johns Hopkins University, USA; 3 Taibah university, Almadinah Almounourah, KSA; 4 Norris Cancer Center, University of Southern California, USA; 5 Department of Pathology, Children's Hospital Los Angeles, Los Angeles, CA, USA

## Background

The search for synergistic drug combinations is critical to the treatment of drug resistant cancers, such as acute myelogenous leukemia (AML), which has 50% to 60% overall survival in children and lower in adults [1]. Conventional treatments include the S phase-specific polymerase II inhibitor 1-h-D-Arabinofuranosylcytosine (ARA-C), but DNA methyltransferase inhibitors such as 5-Aza-2´-deoxycytidine (DAC) also have activity in AML, through DNA genomic demethylation and altered gene expression [2]. Although genome-wide changes in RNA expression associated with DAC and ARA-C are not fully understood [3], characterizing these responses is a critical step to increasing the efficacy of combinatorial therapies that include DAC and ARA-C.

## Materials and methods

RNA expression levels were assessed using the Human Exon 1.0ST (Affymetrix) array 72 hours after single-dose treatments with 1.0 µM DAC or ARA-C in UT7epo, Molm13, and NB4 cells. The half maximal effective concentration (EC50) of DAC and ARA-C was determined in each cell line, and the drug combination index (CI) was calculated using the Chou-Talalay method.

## Results

While increased RRM2 expression is observed following ARA-C exposure and is associated with ARA-C resistance in UT7epo cells, pre-treatment with DAC restores cellular sensitivity and correlates with increased RNA expression of the nucleoside transporter, SLC29A1/hENT1. In contrast, DAC/ARA-C combinations are antagonistic in Molm13 cells, where each drug independently increases RNA expression of C-KIT and associated proliferation pathways.

## Conclusions

Pre-treatment with DAC can sensitize ARA-C resistant AML cells, but can increase resistance in ARA-C sensitive AML cells. Possible molecular mechanisms for these results are suggested by the transcriptional response to DAC. These results show that responses to drug combinations are driven by the molecular responses of individual cell types. The results also provide an alternative approach for predicting what combinations, dosing and scheduling of drug delivery should be used to better individualize therapy.
